# Associations of Demographics, Dependence, and Biomarkers With Transitions in Tobacco Product Use in a Cohort of Cigarette Users and Dual Users of Cigarettes and E-cigarettes

**DOI:** 10.1093/ntr/ntac207

**Published:** 2022-08-29

**Authors:** Fatema Shafie-Khorassani, Megan E Piper, Douglas E Jorenby, Timothy B Baker, Neal L Benowitz, Todd Hayes-Birchler, Rafael Meza, Andrew F Brouwer

**Affiliations:** Department of Biostatistics, University of Michigan, Ann Arbor, MI, USA; Department of Medicine, University of Wisconsin, Madison, WI, USA; Department of Medicine, University of Wisconsin, Madison, WI, USA; Department of Medicine, University of Wisconsin, Madison, WI, USA; Department of Medicine, University of California San Francisco, San Francisco, CA, USA; Department of Medicine, University of Wisconsin, Madison, WI, USA; Department of Epidemiology, University of Michigan, Ann Arbor, MI, USA; Department of Epidemiology, University of Michigan, Ann Arbor, MI, USA

## Abstract

**Introduction:**

It is uncertain whether e-cigarettes facilitate smoking cessation in the real world. We aimed to understand whether and how transitions among cigarette, e-cigarette, and dual use are associated with sociodemographics, dependence measures, and biomarkers.

**Aims and Methods:**

We followed 380 adult daily cigarette users and dual users every 2 months for up to 2 years. We estimated transition rates between noncurrent, cigarette-only, e-cigarette-only, and dual use states using a multistate transition model. We estimated univariable hazard ratios (HR) for demographics, dependence measures for cigarettes and e-cigarettes, biomarkers, spousal or partner behaviors, and other measures.

**Results:**

We estimated that participants transitioned from cigarette-only to e-cigarette-only through a period of dual use. Dual users ceased smoking (transitioning to e-cigarette-only use) at a greater rate than cigarette-only users did (HR 2.44, 95% CI: 1.49, 4.02). However, of the 60% of dual users estimated to transition to single product use in 1 year, 83% would transition to cigarette-only use and only 17% to e-cigarette-only use. E-cigarette dependence measures were generally associated with reduced e-cigarette cessation rather than enhanced cigarette cessation. E-cigarette users motivated by harm or toxicity reduction or because of restrictions on where or when they could smoke had reduced rates of smoking relapse. Cigarette dependence and spousal smoking were barriers to cigarette cessation for dual users, while using e-cigarettes first in the morning, motivation to quit smoking, and sensory, social, and emotional enjoyment of e-cigarettes (secondary dependence motives) were facilitators of smoking cessation among dual users.

**Conclusions:**

Tobacco control policy and interventions may be informed by the barriers and facilitators of product transitions.

**Implications:**

Although e-cigarettes have the potential to promote smoking cessation, their real-world impact is uncertain. In this cohort, dual users were more likely to quit smoking than cigarette-only users, but the overall impact was small because most dual users returned to cigarette-only use. Moreover, e-cigarette dependence promoted continued dual use rather than smoking cessation. Yet, high motivation to quit smoking and the sensory, social, and emotional enjoyment of e-cigarettes facilitated smoking cessation in dual users. Better understanding the barriers and facilitators of transitions can help to develop regulations and interventions that lead to more effective use of e-cigarettes for smoking cessation.

## Introduction

E-cigarettes have been promoted as lower-risk alternatives to combustible tobacco products: They would reduce tobacco-related disease risk and enhance public health if they were to facilitate smoking cessation, as has been found in some clinical trials and other studies.^1–4^ However, e-cigarettes may also represent a public health threat if they serve as a catalyst for youth nicotine addiction and do not meaningfully promote—or even impede—smoking cessation in the real world.^5–7^ Because the introduction of e-cigarettes has changed the landscape of tobacco product use in the United States and other high-income countries^8,9^ and has been promoted as a smoking cessation tool, it is essential to understand the factors that influence whether or not e-cigarette use actually leads to cigarette cessation.

One approach to exploring the effects of e-cigarettes is to use longitudinal data on individual patterns of tobacco product use to identify variables that are associated with product use transitions, for example, from dual use of cigarettes and e-cigarettes to exclusive e-cigarette use. Multistate transition modeling is a statistical modeling approach that can be used to estimate underlying transition rates between different patterns of use. It is increasingly being used in the tobacco control field to analyze longitudinal data.^10–18^ However, previous applications have used data with a relatively long time between follow-ups (typically 1 year). The Exhale study^19^ followed a cohort of 422 cigarette smokers and dual users (ie, users of both cigarettes and e-cigarettes) every 2 months for 2 years, offering a much richer profile of the short-term dynamics of tobacco product use. Here, we go beyond the previous broad assessment of transitions in the first year of the Exhale cohort^20^ by using a multistate transition model framework to estimate underlying transition rates among cigarette, e-cigarette, dual use, and noncurrent use states and to estimate how transition rates differ by demographic, tobacco product use behavior, markers of cigarette and e-cigarette dependence, and tobacco use biomarkers. This work is intended to reveal factors associated with transitions in cigarette and e-cigarette use, which may inform the development of tobacco control policies and cessation interventions.

## Methods

### Study Population

We used data from the Exhale study, a longitudinal cohort study of 422 adult daily cigarette smokers and dual users of cigarettes and e-cigarettes.^19^ Participants were recruited from the Madison and Milwaukee, Wisconsin areas between October 2015 and July 2017, through television and social media advertisements and provided written informed consent. Participants had to be at least 18 years old, able to read and write English, not currently trying to quit cigarette or e-cigarette use, and not currently in treatment for psychosis or bipolar disorder. They also had to be either exclusive cigarette users (smoking ≥5 cigarettes per day for the past 6 months and no e-cigarette use in the past 3 months) or dual users of cigarettes and e-cigarettes (defined as smoking daily and using nicotine-containing e-cigarettes at least once a week for the past 3 months). Participants were followed every 2 months for 2 years. At baseline, data were collected on demographics, smoking and vaping behaviors, cigarette and e-cigarette dependence, and use history. Every 4 months participants had an in-person visit and were provided a urine sample for biomarker measurement. Two months between in-person visits, participants were contacted for phone assessments of product use. We classified participants into one of four use states at each 2-month time point, based on self-reported product use in the last 30 days: Noncurrent user, cigarette-only user, e-cigarette-only user, and dual user. Self-reported abstinence was not biochemically confirmed, but biomarker histograms by state support self-reports overall (Supplementary Figures S1–S3).

### Variable Definitions

Categorization of continuous variables was based on the distribution of responses and the results of exploratory analyses. For the sake of brevity, the categorizations are given in the supplementary material. The demographic variables of interest included age, sex, race and ethnicity, education, any self-reported psychiatric history, and indicators of whether the participant lived with a spouse or partner who smoked or vaped. We evaluated three biomarkers: NNAL (4-(methylnitrosamino)-1-(3-pyridyl)-1-butanol)—a measure of combustible tobacco product smoke exposure—and cotinine and 3HC (3-hydroxycotinine)—two measures of total daily nicotine exposure from all products.

The cigarette behavior and dependence variables included motivation to quit cigarettes, cigarettes per day, Fagerstrom Test for Cigarette Dependence (FTCD) score,^21,22^ time to the first cigarette, Wisconsin Inventory of Smoking Dependence Motives (WISDM) Primary Dependence Motives (PDM) score (a measure of physiological dependence and the compulsion to use^23^), WISDM Secondary Dependence Motives (SDM) score (a measure of social, sensory, and emotional enjoyment and instrumental use),^23^ and WISDM total score.

The e-cigarette behavior and dependence variables included motivation to quit e-cigarettes, flavor, nicotine content, e-cigarette type, frequency of use, e-FTCD score^24^; time to first e-cigarette, e-WISDM^24^ PDM, e-WISDM SDM, e-WISDM total score, and first product used in the morning. We also examined reasons for e-cigarette use, comparing individuals who rate a potential reason as somewhat to extremely important vs not at all important.

### Transition Modeling

We used a Markov multistate transition model to estimate the transition hazard rates between the four states. A Markov multistate transition model is a continuous-time, finite-state stochastic process that assumes the probability of transition depends only on a user’s current state and not their past trajectory of use.^12^ We only observe an individual’s state every 2 months, but the model allows transitions to happen at any point between visits, estimating the instantaneous rate (hazard) of transitioning from one state to another. Transitions hazard rate and hazard ratio were estimated using the MSM package in R (v4.1).^25^

#### Model Reduction

We used a Schwarz Information Criterion to determine the model transition structure.^12^ Here, we disallowed direct, instantaneous transitions from noncurrent use to e-cigarette-only use, from cigarette-only use to e-cigarette-only use or vice versa, and from dual use to noncurrent use (Supplementary Table S2). Disallowing instantaneous transitions does not mean that these transitions cannot occur between observations; rather, it means that an individual must transition through a different (unobserved) state first (eg, transitioning from cigarette-only use to e-cigarette-only use by first transitioning through dual use). In addition, some transitions were too infrequent for us to power estimates of the relative transition rates between covariate groups (ie, hazard ratios). Therefore, we only include hazard ratio results for the following transitions: cigarette-only to noncurrent use, cigarette-only to dual use, e-cigarette-only to dual use, dual use to cigarette-only use, and dual use to e-cigarette-only use.

#### Estimating Transition Hazards, Transition Hazard Ratios, and Covariate Hazard Ratios

We estimated transition rates (hazards) for the allowed instantaneous transitions and cumulative probabilities of transitioning between states after two months and after 1 year. The 2-month cumulative transition probabilities allow for a direct comparison of modeled transitions to observed transitions in the data, while the 1-year cumulative transition probabilities are more comparable to other studies.

We estimated transition hazard ratios, that is, the ratio of transitions hazards for pairs of transition, such as the ratio of the rate from cigarette-only to noncurrent use versus that of dual to e-cigarette-only use or the rate from dual to e-cigarette-only use versus that of dual to cigarette-only use. This allowed us to compare, for example, if cigarette-only users or dual users cease cigarette use at a greater rate, or if dual users were more likely to transition to cigarette-only or e-cigarette-only use.

We estimated covariate hazard ratios for each of the previously described covariates on each transition, that is, the ratio of transition hazards for different levels of a covariate for the same transition. This analysis accounts for longitudinal changes in covariate levels. The effects of cigarette or e-cigarette behavior and dependence measures were included only on transitions from states that included current cigarette or e-cigarette use. The HR were estimated in unadjusted (univariable) models. Participants with missingness for a covariate were excluded from the analysis of that covariate. Finally, we calculated the ratio of the dual to e-cigarette-only transition rate to the dual to cigarette-only transition rate for each covariate.

## Results

### Descriptive Statistics

**Figure 1.**
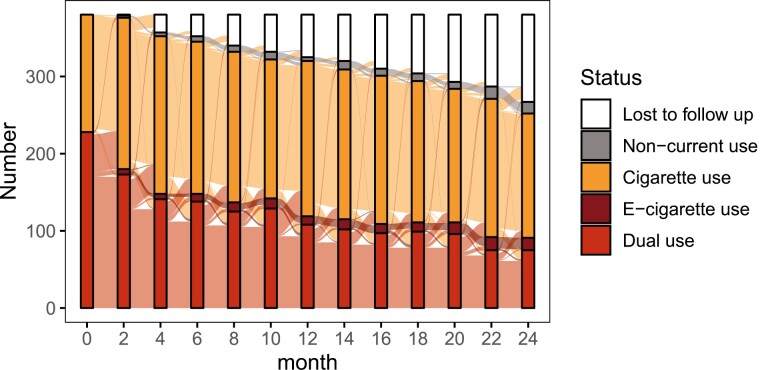
Observed participant tobacco use states over time in the Exhale study.

Of the 422 baseline participants, 380 (90%) completed at least two visits and were included in the transition analyses, and 267 (63%) completed the 2-year assessment. Among these participants, the median number of visits was five. An alluvial plot visualizing the proportion of individuals in each state and transitions among these states between the 2-month follow-up visits is given in Figure 1. Table 1 shows descriptive characteristics of this sample at study baseline. There were 152 (40%) sole cigarette users and 228 (60%) dual users. Characteristics of those who lost to follow up are given in Supplementary Table S3.

**Table 1.** Characteristics of the Exhale Cohort Overall and By Tobacco Use Status At Study Baseline

	Overall (*N* = 380)		Sole cigarette users (*N* = 152)		Dual cigarette and e-cigarette users (*N* = 228)	
	%/mean	N/sd	%/mean	N/sd	%/mean	N/sd
Sex (missing = 0)						
Female	48%	182	51%	77	53%	121
Male	52%	198	49%	75	47%	107
Age (missing = 1)						
18–29	25%	94	19%	29	29%	65
30–49	45%	171	46%	70	44%	101
≥50	30%	114	34%	52	27%	62
Race (missing = 0)						
White	64%	242	53%	81	71%	161
Black	22%	85	36%	54	14%	31
Other	14%	53	11%	17	16%	36
Ethnicity (missing = 14)						
Non-Hispanic	91%	347	93%	141	90%	206
Hispanic	5%	19	3%	5	6%	14
Education (missing = 1)						
More than high school	53%	202	45%	69	58%	133
High school or GED	25%	95	34%	51	19%	44
Less than high school	8%	30	11%	16	6%	14
Age <25	14%	52	11%	16	16%	36
Self-reported psychiatric history (missing = 0)						
Any	53%	202	43%	65	60%	137
None	47%	178	57%	87	40%	91
Lives with spouse or partner who						
Uses cigarettes (missing = 1)	33%	126	34%	52	32%	74
Uses e-cigarettes (missing = 5)	11%	45	1%	2	19%	43
Cigarette dependence measures						
Smoke within 30 min of waking (missing = 1)	72%	274	80%	121	67%	153
CPD (missing = 4)	13.6	8.5	15.6	9.8	12.4	7.3
FTCD (missing = 2)	4.4	2.4	4.8	2.2	4.1	2.5
WISDM total (missing = 4)	46.5	13.3	47.3	13.7	46.0	13.1
WISDM PDM (missing = 2)	3.5	1.5	4.6	1.5	4.4	1.5
WISDM SDM (missing = 4)	4.1	1.2	4.1	1.2	4.1	1.2
Motivation to quit smoking (missing = 3)	3.6	1.7	3.4	1.8	3.7	1.7
E-cigarette dependence measures						
Vape within 30 min of waking (missing = 68)	—	—	—	—	32%	74
VPD (missing = 68)	—	—	—	—	9.9	13.5
E-FTCD (missing = 67)	—	—	—	—	2.6	2.2
E-WISDM total (missing = 66)	—	—	—	—	31.5	13.5
E-WISDM PDM (missing = 66)	—	—	—	—	2.7	1.5
E-WISDM SDM (missing = 66)	—	—	—	—	3.0	1.2
Motivation to quit vaping (missing = 66)	—	—	—	—	2.6	1.8
First product used in the morning (missing = 12)						
Cigarette (100% of the time)	—	—	—		42%	96
Cigarette (>50% of the time)	—	—	—		35%	79
E-cigarette (≥50% of time)	—	—	—		18%	41

CPD = cigarettes per day; FTCD = Fagerstrom Test of Cigarette Dependence; GED = General Educational Development test; PDM = Primary Dependence Measures; SDM = Secondary Dependence Measures; VPD = vaping events per day; WISDM = Wisconsin Inventory of Smoking Dependence Measures. Motivation to quit is a 1–7 scale with 1= not at all motivated and 7 = extremely motivated.

### Transition Probabilities

**Figure 2.**
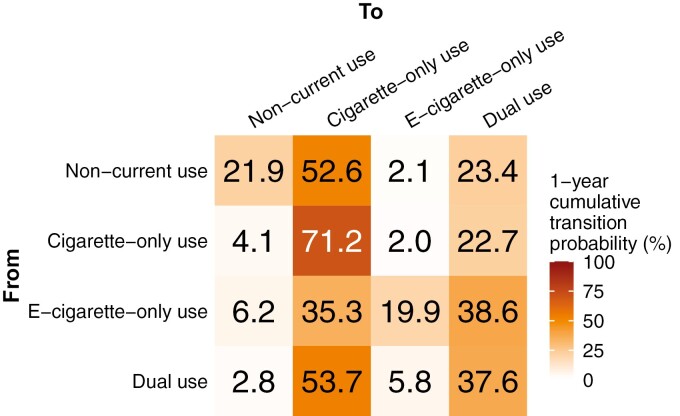
Modeled 1-year cumulative transitions probabilities.

A comparison of the observed and modeled 2-month transition probabilities, demonstrating the model fits the data well, is given in Supplementary Figure S4. In Figure 2, we present the modeled 1-year cumulative transition probabilities, which are more interpretable and comparable to other studies. (A sensitivity analysis removing those who lost to follow up is given in Supplementary Figure S5). The transition rates, cumulative transition probabilities for 2 months and 1 year, and all confidence intervals are given in the supplementary material (Supplementary Table S4). We found that cigarette use was persistent. The model estimated that 93.8% of cigarette-only users would remain cigarette users after 1 year (Figure 2), either as cigarette-only users (71.2%; 95% CI: 67.8, 74.4%) or as part of dual use (22.7%; 95% CI: 19.6, 25.6%). Furthermore, the model estimated that 91.3% of dual users would remain cigarette users, either as cigarette-only users (53.7%; 95% CI: 49.9, 57.7%) or as part of dual use (37.5%; 95% CI: 33.9, 41.4%). In contrast, we found that e-cigarette use was comparatively transient. The model estimated that 58.5% of e-cigarette-only users would remain e-cigarette users, either as e-cigarette-only users (19.9%; 95% CI: 11.3, 34.2%) or as part of dual use (38.6%; 95% CI: 31.8, 44.1%). The model estimated that 43.4% of dual users would remain e-cigarette users, either as e-cigarette-only users (5.8%; 95% CI: 4.1, 8.0%) or as part of dual use (37.6%; 95% CI: 33.9, 41.4%). Finally, the model estimated that participants not using cigarettes (ie, noncurrent or e-cigarette-only users) would quickly return to a cigarette-using category (76.0% of noncurrent users and 59.6% of e-cigarette-only users).

### Transition Hazard Ratios

About 60% of dual users were estimated to transition to sole product use within 1 year, 90% to cigarette-only use and 10% to e-cigarette-only use. Dual users ceased cigarette use (transitioning to e-cigarette-only use) at a greater rate than cigarette-only users transitioned to noncurrent use (HR 2.44, 95% CI: 1.49, 4.02). Dual users also dropped e-cigarette use (transitioning to cigarette-only use) at a greater rate than e-cigarette-only users (HR 4.68, 95% CI: 1.45, 15.1). The difference in transition rate for restarting cigarette use was not statistically significantly different for e-cigarette-only users compared to noncurrent users (HR 1.48, 95% CI: 0.75, 2.90).

### Covariate Hazard Ratios

Covariate hazard ratios are given in Supplementary Table S5. Older age was associated with lower transition rates overall.

Non-Hispanic Black participants had a lower rate of transitioning from cigarette-only to dual use (HR 0.53, 95% CI: 0.35, 0.82) and a greater rate of transitioning from dual to cigarette-only use (HR 3.27, 95% 2.36, 4.52). Having at least a high school education was associated with lower rates of transitioning from e-cigarette-only to dual use. Psychiatric history was associated with cigarette cessation (HR 2.71, 95% CI: 1.25, 5.86), transition from cigarette-only to dual use (HR 1.54, 95% CI: 1.12, 2.11), and dual to e-cigarette-only use (HR 2.75, 95% CI: 1.39, 5.44). Living with a spouse or partner who smokes was associated with a lower rate of transitioning from dual to e-cigarette-only use (HR 0.31, 95% CI: 0.15, 0.66) compared to living with a spouse or partner that does not smoke. On the other hand, living with a spouse or partner who vapes was associated with increased cigarette cessation (HR 2.82, 95% CI: 1.15, 6.91), increased rate of transitioning from cigarette-only to dual use (HR 1.79, 95% CI: 1.04, 3.10), and a reduced rate of transitioning from dual to cigarette-only use (HR 0.64, 95% CI: 0.44, 0.92), compared to living with a spouse or partner that does not vape.

Moderate-to-high levels of all three biomarkers were associated with reduced cigarette cessation rates. Compared to low NNAL, moderate-to-high NNAL was also associated with an increased transition rate from dual to cigarette-only (HR 1.39, 95% CI: 1.07, 1.81) and reduced transition rate from dual to e-cigarette-only use (HR 0.32, 95% CI: 0.17, 0.63).

Smoking within 30 minutes of waking was associated with reduced transitions away from cigarette-only or dual use. Cigarette dependence measures were variously associated with different transitions; generally, increased cigarette dependence measures were associated with reduced cigarette cessation rates (from cigarette-only or dual use) and increased transition from dual to cigarette-only use. We highlight that PDM was more associated with reduced transition rates from dual to e-cigarette-only use while SDM was associated with a greater transition rate from dual to cigarette-only use. Motivation to quit smoking, on the other hand, was substantially associated with cigarette discontinuation whether from cigarette-only (HR 5.05, 95% CI: 2.49, 10.2) or dual (3.35, 95% CI: 1.88, 5.97) use.

Vaping within 30 minutes of waking was only associated with a reduced transition rate from dual to cigarette-only use (HR 0.71, 95% CI: 0.56, 0.91), as was vaping every day (HR 0.25, 95% 0.19, 0.34) compared to some days. Analogous to cigarette dependence measures, e-cigarette dependence measures were primarily associated with reduced transition rates from dual to cigarette-only use. A moderate e-PDM score (vs. low) was associated with reduced transition from dual to cigarette-only use, and a moderate e-SDM score (vs. low) was associated with transition from dual to e-cigarette-only use. A high motivation to quit e-cigarettes was associated with transitions from dual to cigarette-only use (HR 1.99, 95% CI: 1.45, 2.73). For dual users, e-cigarette as a first product in the morning on some days was associated with a reduced transition rate to cigarette-only use (HR 0.38, 95% CI: 0.27, 0.53 for 1–49% of days, HR 0.17, 95% CI: 0.09, 0.31 for ≥50% of days) compared to those who always smoked first. Additionally, using an e-cigarette first ≥50% of the time was associated with increased transitions from dual to e-cigarette-only use (HR 5.63, 95% CI: 2.92, 10.8).

Among e-cigarette device characteristics, increased nicotine concentration was associated with reduced transition rates away from dual to either cigarette-only (HR 0.75 for moderate vs. low, 95% CI: 0.56, 0.99) or to e-cigarette-only use (HR 0.50 for moderate vs low, 95% CI 0.25, 0.99). Although this study was not designed to study the effects of flavors in detail, a lack of flavor preference was strongly associated with transitions from e-cigarette-only to dual use (HR 6.74, 95% CI: 1.07, 42.5) and from dual to cigarette-only use (HR 2.43, 95% CI: 1.21, 4.92). Use of disposable e-cigarettes compared to refillable was associated with transitions from dual to cigarette-only use (HR 1.59, 95% CI: 1.04, 2.43). When examining reasons to use e-cigarettes (Supplementary Table S6), reasons relating to health or toxicity of cigarettes were associated with reduced rates of cigarette use relapse among e-cigarette-only users, as was the ability to use e-cigarettes when and where one could not use cigarettes. Nearly all reasons were significantly associated with reduced rates of dual to cigarette-only use.

To highlight covariates associated with a harm reduction outcome, we compared transition rates from dual to e-cigarette-only use to transition rates from dual to cigarette-only use (Supplementary Table S5, rightmost column). Across the whole cohort, the ratio was 0.20 (95% CI: 0.14, 0.27), meaning that the transition pressure was about 5 times greater for a dual user to cease e-cigarette use than to cease cigarette use. Participants were more likely to transition from dual to cigarette-only use if they smoked ≥10 cigarettes per day or if they had a moderate WISDM SDM score. Participants were more likely to transition from dual to e-cigarette-only use if they used e-cigarettes first ≥50% of the days, had a low WSDM PDM score, vaped every day, or had a moderate total e-WISDM score.

## Discussion

We estimated the rates of changing product use patterns among a cohort of cigarettes and dual users and estimated whether sociodemographic, behavioral, dependence, and biomarker variables were associated with these transitions. Consistent with our previous empirical analysis,^20^ e-cigarette use was not as persistent as cigarette use in this cohort. Here, we estimated that 94% of cigarette-only users and 91% of dual users would still be using cigarettes after 1 year. In comparison, we estimate that 59% of e-cigarette-only users and only 39% of dual users would still be using e-cigarettes after 1 year. These results are consistent with results from the largely contemporaneous Population Assessment of Tobacco and Health (PATH) study, which estimated that 90% of cigarette-only users and 86% of dual users would continue using cigarettes while 72% of e-cigarette and 51% of dual users would continue to use e-cigarettes.^12^ While PATH is a nationally representative longitudinal study, the Exhale cohort was limited to daily smokers and dual users who did not intend to quit smoking or vaping. Both the Exhale and PATH studies largely reflect e-cigarette use prior to widespread use of salt-based e-cigarettes.^26,27^ It is uncertain whether the comparative transience of e-cigarette use will continue with the current and next generations of e-cigarettes.

There is a robust debate about the potential effects of e-cigarettes on public health, with arguments encompassing the potential for smoking cessation and harm reduction, potential impact on youth, and many other issues.^2,3,6,28–31^ One facet of the debate reflects uncertainty about the efficacy of e-cigarette use for smoking cessation outside of the clinical trial context.^6,32^ In our previous, broad examination of yearly transitions in this cohort, we observed that dual users were more likely to attain smoking abstinence after 1 year (8%) compared to sole cigarette users (2%).^20^ In this multistate transition model analysis of the full 2-year cohort, we were able to examine transitions with greater temporal precision and found that dual users transitioned to e-cigarette-only use at 2.44 (95% CI: 1.49, 4.02) times the rate at which cigarette-only users transitioned to noncurrent use. This estimate is quite similar to an analogous result from the PATH study (HR 1.9, 95% CI: 1.6, 2.3).^12^ However, dual use was quite transient (with only 37% of dual users remaining so within 1 year), and 90% of dual users who transitioned did so to cigarette-only use rather than to e-cigarette-only use. Accordingly, the overall impact on the population was limited, but it is important to recognize that participants in this study had no intention to quit smoking or vaping at baseline and were using previous-generation e-cigarette devices. This study was not designed to examine the difference in smoking relapse rates for noncurrent versus e-cigarette-only users.

It was of particular interest to identify variables associated with transition from dual use to either cigarette-only or e-cigarette-only use. The first product used in the morning,^33^ an indicator of the relative dependence on cigarettes vs e-cigarettes, was strongly indicative of the direction of transitions from dual use. Vaping first on at least 50% of days had the greatest association of any variable with transitioning to e-cigarette-only use. Moderate-to-high PDM and e-PDM scores for a product (indicative of heavy, automatic use of a product that feels out of control^23,34^) were associated with fewer transitions away from that product, while moderate-to-high SDM and e-SDM scores (indicative of strong instrumental purposes for use such as sensory effects, affect regulation, and cognitive enhancement) were associated with transitions to the sole use of the corresponding product.

The only reasons to use e-cigarettes that reduced relapse of cigarette use among e-cigarette-only users were related to harm or toxicity of cigarettes or the ability to use e-cigarettes when or where one could not use cigarettes. Nearly every reason was associated with reducing the likelihood of a transition from dual to cigarette-only use, but no reason was associated with the transition from dual to e-cigarette-only use. It is not surprising that dual users whose vaping is related to concerns about cigarette harms and smoking restrictions are more likely to remain e-cigarette users; perhaps more surprising is that none of the motivations were associated with actually quitting smoking. Public health messaging and media attention likely impact perceptions of e-cigarettes and motivations to use them. For example, messaging around e-cigarettes in the United States is more negative than in the United Kingdom,^35^ which may result in different uptake and outcomes.

The highest levels of dependence measures often had weaker associations with transitions away from dual use than did moderate levels when both compared to low levels. This result may indicate a propensity of the most dependent users to remain dual users. Similarly, moderate-to-high levels of nicotine in participants’ e-cigarettes were generally associated with a propensity to remain dual users, compared to those using low nicotine e-cigarettes. Although e-cigarette users self-titrate nicotine consumption,^36^ early e-cigarette devices were less efficient nicotine delivery systems or kept e-liquid nicotine concentrations lower because of the unpleasantness of inhaling high concentrations of freebase nicotine,^37,38^ so it is possible that dual users remained cigarette users to attain desired nicotine consumption. Nevertheless, more work is needed to understand whether e-cigarettes that produce increased e-cigarette dependence or greater sensory enjoyment (eg, nicotine-salt-based products^39,40^) increase or decrease smoking cessation rates.

Spouse or partner product use was associated with continued use or transition to use of the spouse or partner’s product (eg, more likely to transition from cigarette-only to dual use if one’s spouse vaped). Such patterns have been reported in other studies.^41,42^ The impact of spousal behavior presents an opportunity for partner or family-based interventions that address the couple’s tobacco-related routines.^43^ Self-reported psychiatric history was associated with an increased transition rate toward e-cigarette-only use, including from dual to e-cigarette-only use. Psychiatric disorders increase the risk of substance use, including the use of tobacco products,^44^ but it is not clear why psychiatric history would be associated with e-cigarette use specifically. As similar associations have been reported previously,^45,46^ more work is needed to understand the ability of e-cigarettes to serve as harm reduction for this population that is at an elevated risk of smoking-related death and disease.^47^

Unlike other longitudinal studies of individual tobacco use patterns, including PATH, follow-up periodicity in the Exhale cohort was every 2 months (compared to once a year, typically), affording greater opportunity to assess the short-term dynamics of product use. The multistate transition model approach, which is increasingly being used in the tobacco control field (eg,^10–18^), is another strength of this work because it provides a framework for estimating competing transition rates as well as transition hazard ratios for variables of interest. However, this approach is limited in its reliance on participants’ current states as opposed to their longer-term histories (eg, observations of multiple quit attempts) when estimating most likely future states. Another limitation is that the sample was not population based.

This work highlights the determinants of transitions between different patterns of nicotine product use and highlights barriers to and facilitators of the use of e-cigarettes to stop cigarette use, which may lead to more effective tobacco control strategies. Spouse or partner product use may be a barrier to cessation of the spouse’s product, and high cigarette dependence may impede smoking cessation regardless of e-cigarette use. The factors that were highly associated with transition from dual to e-cigarette-only use were using e-cigarettes as the first product in the morning (a marker of e-cigarette dependence), motivation to quit cigarettes, and moderate e-cigarette WISDM SDM scores, indicating e-cigarette user enjoyed the sensory, social, and emotional aspects of e-cigarette use. Overall, the results suggest that transitions reflect a mosaic of social, contextual, and personal factors, with relative levels of product dependence and product reward being especially important.

## Supplementary Material

A Contributorship Form detailing each author’s specific involvement with this content, as well as any supplementary data, are available online at https://academic.oup.com/ntr.

ntac207_suppl_Supplementary_Material

ntac207_suppl_Supplementary_Taxonomy-form

## Data Availability

Data may be available from the corresponding author upon reasonable request. IRB approval and a DUA may be required.
