# Supplementary material for: Associations of Demographics, Dependence, and Biomarkers With Transitions in Tobacco Product Use in a Cohort of Cigarette Users and Dual Users of Cigarettes and E-cigarettes
Source: Nicotine Tob Res. 2022 Aug 29;25(3):462–9. doi: 10.1093/ntr/ntac207 (PMC9910158; doi:10.1093/ntr/ntac207)
Supplement: ntac207_suppl_Supplementary_Material [file ntac207_suppl_supplementary_material.docx]

Supplementary material for “Association of demographics, dependence, and biomarkers with transitions in tobacco product use in a cohort of cigarette users and dual users of cigarettes and e-cigarettes”

**Table S1. Categorization of variables in this analysis of the Exhale study.**

| **Variable** | **Categorization** |
| --- | --- |
| Age | 18-29, 30-49, 50+ years |
| Sex | Male, female |
| Race | Black, White, Other |
| Ethnicity | Hispanic, non-Hispanic |
| Education* | < High school, high school or equivalent, > high school |
| Psychiatric history | None/any self-reported history of depression, bipolar, schizophrenia, anxiety, panic, post-traumatic stress disorder (PTSD) |
| Lives with spouse that smokes/vapes | Yes/no/did not live with a partner |
| Biomarkers | Low or moderate/high based on the lowest tertile of the logarithm of the observed values |
| Motivation to quit cigarettes | Low (1–5), high (6–7) |
| Cigarettes per day | Low (<10 per day), high (10+ per day) |
| FTCD | Low (<5), high (5+) |
| Time to first cigarette | <30 min, ≥30 min |
| WISDM PDM | Low (<3), moderate (3 to <5), high (5+) |
| WISDM SDM | Low (<3), moderate (3 to <5), high (5+) |
| WISDM total | Low (<40), moderate (40–54), high (55+) |
| Motivation to quit e-cigarettes | Low (1–5), high (6–7) |
| Frequency of vaping | Everyday, someday use |
| e-FTCD | Low (<5), high (5+) |
| Time to first e-cigarette | *<*30 min, ≥30 min |
| e-WISDM PDM | Low (<3), moderate (3 to <5), high (5+) |
| e-WISDM SDM | Low (<3), moderate (3 to <5), high (5+) |
| e-WISDM total | Low (<40), moderate (40–54), high (55+) |
| E-cigarette flavor | Tobacco or unflavored, other flavor, no preference |
| E-cigarette nicotine content | Low (0–6 mg), medium (7–17 mg), high (18+ mg) |
| E-cigarette device | Disposable, replaceable, refillable, other |
| First product used in the morning | Cigarettes 100% of the times, cigarettes *>*50% of the time, e-cigarettes ≥50% of the time |

*Only defined for participants ≥25 years of age since younger participants may still be in the education system


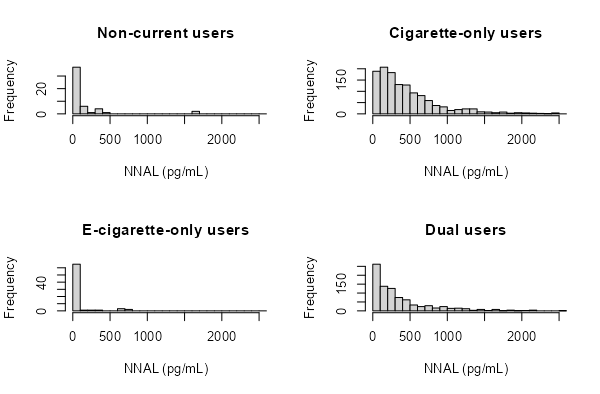


**Figure S1. Histogram of NNAL by self-reported product use.**


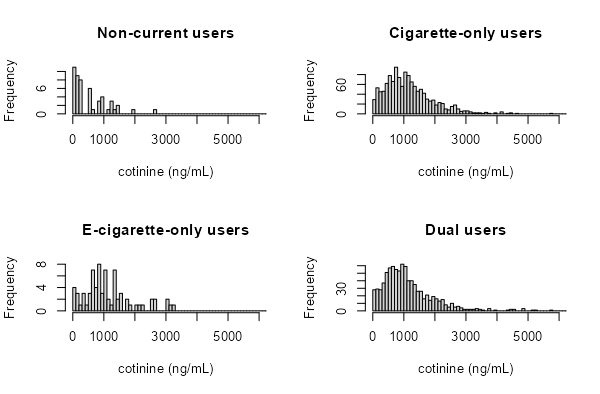


**Figure S2. Histogram of cotinine by self-reported product use.**


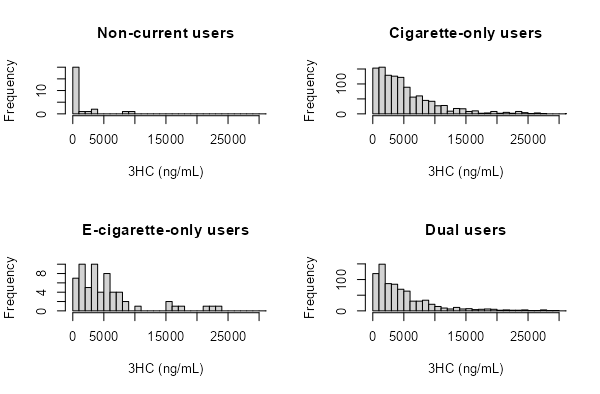


**Figure S3. Histogram of 3HC by self-reported product use.**

**Model reduction**

The table of states observed between pairs of transitions is given below, with initial states listed on the left, and subsequent states above.

**Table S2. Number of observations of each pair states between subsequent visits.**

|  | Non-current use | Cigarette-only use | E-cigarette-only use | Dual use |
| --- | --- | --- | --- | --- |
| Non-current use | 69 | 14 | 2 | 5 |
| Cigarette-only use | 26 | 1980 | 3 | 159 |
| E-cigarette-only use | 2 | 2 | 97 | 27 |
| Dual use | 11 | 259 | 43 | 1167 |

We can see that some transitions, e.g., cigarette-only use to e-cigarette only use are rarely observed and thus could potentially be removed from the model. However, some *direct* transitions may be negligible even if they are observed between subsequent time points, if there is a possible fast indirect set of transitions between them. Accordingly, we use a Schwarz Information Criterion (SIC) to test whether each pathway can be removed without substantially reducing the fit of the model. The SIC balances model parsimony (number of parameters) with model fit.

SIC = log(number of possible transitions) * number of model parameters – 2* model log-likelihood

In the full model, SIC = 3738.8 and in the reduced model, SIC = 3721.6. The SIC of the reduced model is lower, indicating that removing the negligible transitions improved model parsimony without adversely affecting the model fit.


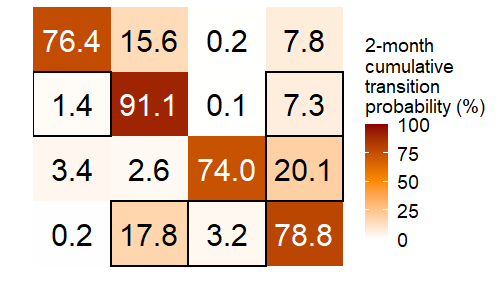

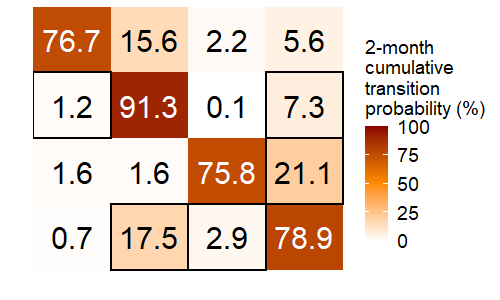


E-cigarette use (sole)

Dual use

**From**

Cigarette use (sole)

a) Observed 2-month cumulative transition probabilities

Non-current use

Cigarette use (sole)

E-cigarette use (sole)

Dual use

Non-current use

**To**

E-cigarette use (sole)

Dual use

**From**

Cigarette use (sole)

b) Modeled 2-month cumulative transition probabilities

Non-current use

**To**

Non-current use

Cigarette use (sole)

E-cigarette use (sole)

Dual use

**Figure S4. Transition probability heatmaps of a) the observed 2-month cumulative transition probabilities and b) the modeled 2-month cumulative transition probabilities.** The boxes highlight the transitions of particular interest.

**Analysis of loss to follow-up**

Below we compare the characteristics of those that completed the 2 years of the study and those that were lost to follow up before the end of the study. Participants who were lost-to-follow-up were more likely to be male, younger, and white.

**Table S3. Comparison of the sociodemographic characteristics of the Exhale cohort overall and between those that complete the study vs those that were lost-to-follow-up before the end of the study.** Abbreviations: GED=General Educational Development test.

|  | Overall  (N=380) | | Participants who completed the study  (N=267) | | Participants who were lost-to-follow-up  (N=113) | |
| --- | --- | --- | --- | --- | --- | --- |
|  | %/mean | N/sd | %/mean | N/sd | %/mean | N/sd |
| Sex (missing = 0) |  |  |  |  |  |  |
| Female | 48% | 182 | 50% | 133 | 43% | 49 |
| Male | 52% | 198 | 50% | 134 | 57% | 64 |
| Age (missing = 1) |  |  |  |  |  |  |
| 18-29 | 25% | 94 | 20% | 54 | 35% | 40 |
| 30-49 | 45% | 171 | 44% | 118 | 47% | 53 |
| 50+ | 30% | 114 | 35% | 94 | 18% | 20 |
| Race (missing = 0) |  |  |  |  |  |  |
| White | 64% | 242 | 61% | 164 | 69% | 78 |
| Black | 22% | 85 | 25% | 67 | 16% | 18 |
| Other | 14% | 53 | 13% | 36 | 15% | 17 |
| Ethnicity (missing = 14) |  |  |  |  |  |  |
| Non-Hispanic | 91% | 347 | 90% | 241 | 94% | 106 |
| Hispanic | 5% | 19 | 6% | 15 | 4% | 4 |
| Education (missing = 1) |  |  |  |  |  |  |
| More than high school | 53% | 202 | 54% | 143 | 52% | 59 |
| High school/GED | 25% | 95 | 25% | 68 | 24% | 27 |
| Less than high school | 8% | 30 | 9% | 25 | 4% | 5 |
| Age <25 | 14% | 52 | 11% | 30 | 19% | 21 |

As a sensitivity analysis, we compute the overall transitions for the 267 individuals who completed the full 2 years of the study. This figure is analogous to Figure 2 in the main text. The estimated transition probabilities are comparable.

**Figure S5: Modeled 1-year cumulative transitions probabilities for participants completing the full study.**

**Table S4. Point estimates and 95% confidence intervals for the transition hazards and one-year transition probabilities.**

|  | Transition hazard  (per month) | | Two-month transition probability (%) | | One-year transition probability (%) | |
| --- | --- | --- | --- | --- | --- | --- |
| Transition | Estimate | 95% CI | Estimate | 95% CI | Estimate | 95% CI |
| Non-current use to |  |  |  |  |  |  |
| Non-current use | -0.135 | (-0.208, -0.088) | 76.4 | (67.0, 85.6) | 21.9 | (11.2, 39.4) |
| Cigarette use | 0.089 | (0.051, 0.155) | 15.6 | (8.3, 23.1) | 52.6 | (38.9, 62.8) |
| E-cigarette use | — | — | 0.2 | (0.0, 0.3) | 2.1 | (1.0, 3.4) |
| Dual use | 0.047 | (0.021, 0.104) | 7.8 | (2.7, 13.9) | 23.4 | (14.6, 30.8) |
| Cigarette use to |  |  |  |  |  |  |
| Non-current use | 0.009 | (0.006, 0.012) | 1.4 | (0.9, 2.0) | 4.1 | (2.7, 5.9) |
| Cigarette use | -0.052 | (-0.059, -0.044) | 91.1 | (89.9, 92.3) | 71.2 | (67.8, 74.4) |
| E-cigarette use | — | — | 0.1 | (0.1, 0.2) | 2.0 | (1.4, 2.8) |
| Dual use | 0.043 | (0.037, 0.050) | 7.3 | (6.2, 8.4) | 22.7 | (19.6, 25.6) |
| ENDS use to |  |  |  |  |  |  |
| Non-current use | 0.023 | (0.007, 0.072) | 3.4 | (0.0, 8.0) | 6.2 | (0.9, 12.5) |
| Cigarette use | — | — | 2.6 | (1.7, 3.4) | 35.3 | (27.8, 41.7) |
| E-cigarette use | -0.154 | (-0.219, -0.108) | 74.0 | (65.6, 81.8) | 19.9 | (11.3, 34.2) |
| Dual use | 0.131 | (0.090, 0.190) | 20.1 | (13.6, 26.9) | 38.6 | (31.8, 44.1) |
| Dual use to |  |  |  |  |  |  |
| Non-current use | — | — | 0.2 | (0.1, 0.3) | 2.8 | (1.9, 4.1) |
| Cigarette use | 0.106 | (0.093, 0.119) | 17.8 | (15.9, 19.8) | 53.7 | (49.9, 57.7) |
| E-cigarette use | 0.021 | (0.016, 0.028) | 3.2 | (2.3, 4.1) | 5.8 | (4.1, 8.0) |
| Dual use | -0.127 | (-0.142, -0.113) | 78.8 | (76.6, 80.8) | 37.6 | (33.9, 41.4) |

**Table S5. Covariate hazard ratios (HR) and 95% confidence intervals (CI) for selected transitions between tobacco use states.** Bold values in the first five columns indicate that the CI does not include 1; bold values in the final column indicate statistically significant difference from the full population estimate (HR 0.20, 95% CI 0.14, 0.27). Abbreviations: GED=General Educational Development test; FTCD=Fagerstrom Test of Cigarette Dependence; WISDM=Wisconsin Inventory of Smoking Dependence Measures; PDM=Primary Dependence Measures; SDM=Secondary Dependence Measures. †Product use by spouse was assessed separately, e.g., a spouse that used cigarettes but not e-cigarettes would be categorized as a “Yes” for the cigarette category and as a “No, spouse/partner does not use” for the e-cigarette category, and vice versa.

|  | | Cigarette-only to non-current use  (N= 26) | | Cigarette-only to dual use  (N=159) | | E-cigarette-only to dual use  (N=27) | | Dual to cigarette-only use  (N=259) | | Dual to e-cigarette-only use  (N=43) | |  | Dual to e-cigarette-only vs dual to cigarette-only | |
| --- | --- | --- | --- | --- | --- | --- | --- | --- | --- | --- | --- | --- | --- | --- |
|  | | HR | 95% CI | HR | 95% CI | HR | 95% CI | HR | 95% CI | HR | 95% CI |  | HR | 95% CI |
| **Demographics** | |  |  |  |  |  |  |  |  |  |  |  |  |  |
| Sex | Female | 1.00 | (ref) | 1.00 | (ref) | 1.00 | (ref) | 1.00 | (ref) | 1.00 | (ref) |  | 0.19 | (0.12, 0.30) |
|  | Male | 0.69 | (0.31, 1.55) | 1.08 | (0.79, 1.48) | 0.90 | (0.42, 1.95) | 1.05 | (0.82, 1.34) | 1.19 | (0.67, 2.10) |  | 0.22 | (0.14, 0.34) |
| Age | 18-29 | 1.00 | (ref) | 1.00 | (ref) | 1.00 | (ref) | 1.00 | (ref) | 1.00 | (ref) |  | 0.24 | (0.09, 0.60) |
|  | 30-49 | 0.54 | (0.19, 1.47) | 0.72 | (0.49, 1.06) | 0.55 | (0.24, 1.28) | **0.71** | **(0.51, 0.98)** | 0.56 | (0.23, 1.37) |  | 0.19 | (0.12, 0.30) |
|  | 50+ | **0.03** | **(0.00, 0.26)** | **0.46** | **(0.30, 0.70)** | 0.58 | (0.21, 1.58) | **0.61** | **(0.43, 0.87)** | **0.35** | **(0.13, 0.99)** |  | 0.14 | (0.07, 0.28) |
| Race/ethnicity | Non-Hispanic White | 1.00 | (ref) | 1.00 | (ref) | 1.00 | (ref) | 1.00 | (ref) | 1.00 | (ref) |  | 0.27 | (0.19, 0.39) |
|  | Non-Hispanic Black | 0.93 | (0.37, 2.32) | **0.53** | **(0.35, 0.82)** | 0.27 | (0.04, 2.01) | **3.27** | **(2.36, 4.52)** | 0.60 | (0.19, 1.96) |  | 0.05 | (0.02, 0.16) |
|  | Hispanic | 4.05 | (0.70, 23.2) | 0.57 | (0.14, 2.35) | 1.20 | (0.01, 154) | 1.22 | (0.71, 2.08) | 1.90 | (0.64, 5.61) |  | 0.43 | (0.13, 1.41) |
|  | Non-Hispanic Other | 1.51 | (0.48, 4.75) | 1.06 | (0.65, 1.75) | 0.81 | (0.19, 3.45) | 1.47 | (0.99, 2.19) | 0.53 | (0.16 1.74) |  | 0.10 | (0.03, 0.33) |
| Education | More than high school | 0.55 | (0.17, 1.75) | 0.96 | (0.45, 2.07) | **0.12** | **(0.02, 0.81)** | 0.74 | (0.44, 1.27) | 0.44 | (0.07, 2.81) |  | 0.18 | (0.12, 0.27) |
|  | High school/GED | 0.47 | (0.16, 1.41) | 2.32 | (1.17, 4.61) | **0.17** | **(0.04, 0.85)** | 0.96 | (0.60, 1.55) | 0.98 | (0.18, 5.24) |  | 0.11 | (0.04, 0.27) |
|  | Less than high school | 1.00 | (ref) | 1.00 | (ref) | 1.00 | (ref) | 1.00 | (ref) | 1.00 | (ref) |  | 0.18 | (0.03, 0.97) |
| Psychiatric history | Any | **2.71** | **(1.25, 5.86)** | **1.54** | **(1.12, 2.11)** | 2.08 | (0.84, 5.13) | 0.96 | (0.75, 1.23) | **2.75** | **(1.39, 5.44)** |  | 0.28 | (0.19, 0.40) |
|  | None | 1.00 | (ref) | 1.00 | (ref) | 1.00 | (ref) | 1.00 | (ref) | 1.00 | (ref) |  | 0.10 | (0.05, 0.18) |
| Lives with spouse/partner who uses cigarettes† | Yes | 2.32 | (0.87, 6.18) | 1.12 | (0.72, 1.74) | 1.04 | (0.38, 2.86) | 1.20 | (0.85, 1.68) | **0.31** | **(0.15, 0.66)** |  | 0.11 | (0.06, 0.21) |
|  | No, spouse/ partner does not use | 1.00 | (ref) | 1.00 | (ref) | 1.00 | (ref) | 1.00 | (ref) | 1.00 | (ref) |  | 0.42 | (0.26, 0.70) |
|  | Does not live with a spouse/partner | 0.61 | (0.17, 2.13) | 1.25 | (0.45, 1.89) | 3.61 | (0.02, 8.58) | 1.22 | (0.44, 1.69) | 0.62 | (0.07, 1.17) |  | 0.21 | (0.13, 0.36) |
| Lives with spouse/partner who uses e-cigarettes† | Yes | **2.82** | **(1.15, 6.91)** | **1.79** | **(1.04, 3.10)** | 1.28 | (0.36, 4.48) | **0.64** | **(0.44, 0.92)** | 0.51 | (0.22, 1.20) |  | 0.19 | (0.08, 0.42) |
|  | No, spouse/partner does not use | 1.00 | (ref) | 1.00 | (ref) | 1.00 | (ref) | 1.00 | (ref) | 1.00 | (ref) |  | 0.23 | (0.15, 0.36) |
|  | Does not live with a spouse/partner | 0.39 | (0.14, 1.08) | 1.21 | (0.87, 1.69) | 3.71 | (1.65, 8.37) | 0.90 | (0.69, 1.17) | 0.84 | (0.45, 1.57) |  | 0.22 | (0.13, 0.36) |
| **Biomarkers** | |  |  |  |  |  |  |  |  |  |  |  |  |  |
| NNAL | Low | 1.00 | (ref) | 1.00 | (ref) | 1.00 | (ref) | 1.00 | (ref) | 1.00 | (ref) |  | 0.42 | (0.28, 0.64) |
|  | Moderate/high | **0.15** | **(0.07, 0.34)** | 0.72 | (0.51, 1.01) | 2.28 | (0.94, 5.56) | **1.39** | **(1.07, 1.81)** | **0.32** | **(0.17, 0.63)** |  | 0.10 | (0.05, 0.18) |
| Cotinine | Low | 1.00 | (ref) | 1.00 | (ref) | 1.00 | (ref) | 1.00 | (ref) | 1.00 | (ref) |  | 0.24 | (0.14, 0.40) |
|  | Moderate/high | **0.32** | **(0.15, 0.69)** | 0.87 | (0.62, 1.21) | 0.67 | (0.31, 1.42) | 0.88 | (0.68, 1.13) | 0.68 | (0.37, 1.23) |  | 0.18 | (0.12, 0.27) |
| 3HC | Low | 1.00 | (ref) | 1.00 | (ref) | 1.00 | (ref) | 1.00 | (ref) | 1.00 | (ref) |  | 0.16 | (0.08, 0.28) |
|  | Moderate/high | **0.19** | **(0.09, 0.44)** | 0.72 | (0.52, 1.01) | 1.17 | (0.49, 2.79) | 0.95 | (0.73, 1.22) | 1.35 | (0.70, 2.59) |  | 0.22 | (0.15, 0.32) |

| **Cigarette dependence measures** | |  |  |  |  |  |  |  |  |  |  |  |  |  |
| --- | --- | --- | --- | --- | --- | --- | --- | --- | --- | --- | --- | --- | --- | --- |
| Smoke within 30 min of waking | Yes | **0.19** | **(0.08, 0.39)** | **0.68** | **(0.49, 0.94)** | - | - | 0.86 | (0.67, 1.11) | **0.23** | **(0.12, 0.42)** |  | 0.10 | (0.06, 0.16) |
|  | No | 1.00 | (ref) | 1.00 | (ref) | - | - | 1.00 | (ref) | 1.00 | (ref) |  | 0.37 | (0.24, 0.55) |
| Cigarettes per day | Low | 1.00 | (ref) | 1.00 | (ref) | - | - | 1.00 | (ref) | 1.00 | (ref) |  | 0.30 | (0.21, 0.43) |
|  | High | **0.20** | **(0.08, 0.53)** | 1.00 | (0.73, 1.37) | - | - | 1.17 | (0.92, 1.50) | **0.25** | **(0.11, 0.55)** |  | **0.06** | **(0.03, 0.14)** |
| FTCD | Low | 1.00 | (ref) | 1.00 | (ref) | - | - | 1.00 | (ref) | 1.00 | (ref) |  | 0.21 | (0.14, 0.31) |
|  | High | **0.13** | **(0.04, 0.40)** | **0.51** | **(0.37, 0.70)** | - | - | **0.74** | **(0.58, 0.95)** | 0.59 | (0.32, 1.09) |  | 0.17 | (0.10, 0.29) |
| WISDM total | Low | 1.00 | (ref) | 1.00 | (ref) | - | - | 1.00 | (ref) | 1.00 | (ref) |  | 0.30 | (0.19, 0.49) |
|  | Moderate | 0.44 | (0.22, 1.13) | 1.29 | (0.90, 1.86) | - | - | **1.44** | **(1.08, 1.92)** | 0.82 | (0.44, 1.52) |  | 0.17 | (0.11, 0.29) |
|  | High | 0.49 | (0.16, 1.22) | 1.19 | (0.78, 1.81) | - | - | **1.59** | **(1.15, 2.20)** | 0.64 | (0.29, 1.45) |  | 0.12 | (0.06, 0.26) |
| WISDM PDM | Low | 1.00 | (ref) | 1.00 | (ref) | - | - | 1.00 | (ref) | 1.00 | (ref) |  | **0.52** | **(0.30, 0.88)** |
|  | Moderate | 0.34 | (0.15, 0.77) | 1.31 | (0.85, 2.01) | - | - | **1.61** | **(1.15, 2.26)** | **0.33** | **(0.16, 0.66)** |  | 0.10 | (0.06, 0.19) |
|  | High | **0.27** | **(0.10, 0.68)** | 1.05 | (0.67, 1.65) | - | - | 1.39 | (0.98, 1.97) | **0.44** | **(0.22, 0.85)** |  | 0.16 | (0.09, 0.29) |
| WISDM SDM | Low | 1.00 | (ref) | 1.00 | (ref) | - | - | 1.00 | (ref) | 1.00 | (ref) |  | 0.32 | (0.17, 0.60) |
|  | Moderate | 1.25 | (0.51, 3.05) | 1.31 | (0.87, 1.97) | - | - | **1.54** | **(1.10, 2.15)** | 1.12 | (0.59, 2.12) |  | 0.23 | (0.16, 0.35) |
|  | High | 0.82 | (0.27, 2.54) | 1.39 | (0.86, 2.24) | - | - | **2.45** | **(1.69, 3.57)** | 0.43 | (0.14, 1.33) |  | **0.06** | **(0.02, 0.16)** |
| Motivation to quit cigarettes | Low | 1.00 | (ref) | 1.00 | (ref) | - | - | 1.00 | (ref) | 1.00 | (ref) |  | 0.14 | (0.10, 0.21) |
|  | High | **5.05** | **(2.49, 10.2)** | 0.92 | (0.57, 1.50) | - | - | 1.04 | (0.75, 1.50) | **3.35** | **(1.88, 5.97)** |  | 0.47 | (0.27, 0.81) |
| **E-cigarette dependence measures** | |  |  |  |  |  |  |  |  |  |  |  |  |  |
| Vape within 30 min of waking | Yes | - | - | - | - | 0.88 | (0.40, 1.94) | **0.71** | **(0.56, 0.91)** | 1.08 | (0.61, 1.93) |  | 0.24 | (0.16, 0.38) |
|  | No | - | - | - | - | 1.00 | (ref) | 1.00 | (ref) | 1.00 | (ref) |  | 0.16 | (0.10, 0.25) |
| Vaping frequency | Somedays | - | - | - | - | 1.00 | (ref) | 1.00 | (ref) | 1.00 | (ref) |  | 0.09 | (0.06, 0.16) |
|  | Everyday | - | - | - | - | 1.08 | (0.38, 3.08) | **0.25** | **(0.19, 0.34)** | 1.53 | (0.84, 2.80) |  | **0.57** | **(0.37, 0.89)** |
| E-FTCD | Low | - | - | - | - | 1.00 | (ref) | 1.00 | (ref) | 1.00 | (ref) |  | 0.33 | (0.22, 0.50) |
|  | High | - | - | - | - | 1.03 | (0.35, 3.09) | **0.45** | **(0.25, 0.81)** | 0.56 | (0.20, 1.58) |  | 0.41 | (0.13, 1.33) |
| E-WISDM total | Low | - | - | - | - | 1.00 | (ref) | 1.00 | (ref) | 1.00 | (ref) |  | 0.10 | (0.06, 0.17) |
|  | Moderate | - | - | - | - | 2.15 | (0.80, 5.79) | **0.68** | **(0.46, 0.99)** | **3.51** | **(1.71, 7.22)** |  | **0.55** | **(0.28, 1.05)** |
|  | High | - | - | - | - | 1.57 | (0.63, 3.93) | **0.58** | **(0.43, 0.77)** | 1.72 | (0.88, 3.39) |  | 0.31 | (0.18, 0.54) |
| E-WISDM PDM | Low | - | - | - | - | 1.00 | (ref) | 1.00 | (ref) | 1.00 | (ref) |  | 0.18 | (0.12, 0.26) |
|  | Moderate | - | - | - | - | 1.90 | (0.75, 4.84) | **0.53** | **(0.34, 0.83)** | 1.17 | (0.49, 2.80) |  | 0.39 | (0.15, 0.99) |
|  | High | - | - | - | - | 0.62 | (0.18, 2.10) | 0.72 | (0.47, 1.09) | 0.80 | (0.25, 2.58) |  | 0.20 | (0.06, 0.71) |
| E-WISDM SDM | Low | - | - | - | - | 1.00 | (ref) | 1.00 | (ref) | 1.00 | (ref) |  | 0.15 | (0.10, 0.23) |
|  | Moderate | - | - | - | - | **2.94** | **(1.34, 6.44)** | 0.81 | (0.61, 1.07) | **1.97** | **(1.09, 3.55)** |  | 0.37 | (0.22, 0.63) |
|  | High | - | - | - | - | 4.28 | (0.89, 19.6) | 0.60 | (0.32, 1.14) | 0.85 | (0.14, 5.19) |  | 0.22 | (0.03, 1.50) |
| Motivation to quit e-cigarettes | Low | - | - | - | - | 1.00 | (ref) | 1.00 | (ref) | 1.00 | (ref) |  | 0.23 | (0.16, 0.31) |
|  | High | - | - | - | - | 0.47 | (0.11, 2.00) | **1.99** | **(1.45, 2.73)** | 0.38 | (0.09, 1.57) |  | 0.04 | (0.01, 0.18) |
| First product used | Cigarette (100% of time) | - | - | - | - | - | - | 1.00 | (ref) | 1.00 | (ref) |  | 0.08 | (0.05, 0.15) |
|  | Cigarette (>50% of time) | - | - | - | - | - | - | **0.38** | **(0.27, 0.53)** | 0.84 | (0.34, 2.09) |  | 0.19 | (0.09, 0.42) |
|  | E-cigarette (≥50% of time) |  |  |  |  |  |  | **0.17** | **(0.09, 0.31)** | **5.63** | **(2.92, 10.8)** |  | **2.87** | **(1.41, 5.85)** |

| **E-cigarette characteristics** | |  |  |  |  |  |  |  |  |  |  |  |  |  |
| --- | --- | --- | --- | --- | --- | --- | --- | --- | --- | --- | --- | --- | --- | --- |
| Nicotine concentration | Low | - | - | - | - | 1.00 | (ref) | 1.00 | (ref) | 1.00 | (ref) |  | 0.28 | (0.18, 0.42) |
|  | Moderate | - | - | - | - | 1.90 | (0.83, 4.32) | **0.75** | **(0.56, 0.99)** | **0.50** | **(0.25, 0.99)** |  | 0.19 | (0.10, 0.34) |
|  | High | - | - | - | - | 0.48 | (0.14, 1.66) | 0.84 | (0.62, 1.13) | **0.31** | **(0.13, 0.71)** |  | 0.10 | (0.05, 0.22) |
| Preferred flavor | Unflavored/tobacco flavored | - | - | - | - | 1.00 | (ref) | 1.00 | (ref) | 1.00 | (ref) |  | 0.35 | (0.15, 0.84) |
|  | Any other flavor |  |  |  |  | 1.86 | (0.44, 7.91) | **1.93** | **(1.12, 3.31)** | 1.07 | (0.49, 2.32) |  | 0.19 | (0.14, 0.28) |
|  | No preferred flavor | - | - | - | - | **6.74** | **(1.07, 42.5)** | **2.43** | **(1.21, 4.92)** | 1.80 | (0.42, 7.63) |  | 0.26 | (0.07, 0.99) |
| Type | Refillable | - | - | - | - | 1.00 | (ref) | 1.00 | (ref) | 1.00 | (ref) |  | 0.26 | (0.18, 0.38) |
|  | Disposable | - | - | - | - | 0.72 | (0.10, 5.39) | **1.59** | **(1.04, 2.43)** | 0.34 | (0.003,35.4) |  | 0.14 | (0.06, 0.33) |
|  | Replaceable | - | - | - | - | 1.04 | (0.31, 3.49) | 1.21 | (0.89, 1.64) | 0.36 | (0.13, 1.03) |  | 0.08 | (0.03, 0.22) |

**Table S6. Covariate hazard ratios (HR) and 95% confidence intervals (CI) for e-cigarette motivation.** All rows give the HRs for endorsing a motivation as somewhat to extremely important vs not at all important. Bold values indicate that the CI does not include 1.

|  | E-cigarette to dual use (N=27) | | Dual to cigarette-only use  (N=259) | | Dual to e-cigarette-only use (N=43) | |
| --- | --- | --- | --- | --- | --- | --- |
| **Motivation to use e-cigarettes** | HR | 95% CI | HR | 95% CI | HR | 95% CI |
| E-cigarettes might be less harmful to the people around me than regular cigarettes | **0.26** | **(0.10, 0.72)** | **0.27** | **(0.21, 0.35)** | 0.80 | (0.36, 1.77) |
| I am unable to stop using e-cigarettes | 1.00 | (0.47, 2.12) | **0.41** | **(0.30, 0.56)** | 1.49 | (0.84, 2.66) |
| They are affordable | 1.06 | (0.42, 2.66) | **0.35** | **(0.28, 0.45)** | 1.00 | (0.53, 1.87) |
| E-cigarettes are cheaper than smoking | 0.71 | (0.29, 1.69) | **0.37** | **(0.29, 0.47)** | 0.97 | (0.52, 1.80) |
| I can use e-cigarettes at times when or in places where smoking cigarettes isn’t allowed | **0.32** | **(0.11, 0.94)** | **0.33** | **(0.26, 0.43)** | 1.06 | (0.47, 2.39) |
| To help with nicotine withdrawal symptoms when I can’t smoke | 0.62 | (0.27, 1.42) | **0.31** | **(0.24, 0.39)** | 0.73 | (0.39, 1.37) |
| E-cigarettes come in flavors I like | 1.04 | (0.39, 2.76) | **0.45** | **(0.35, 0.57)** | 1.65 | (0.86, 3.15) |
| E-cigarettes might be less harmful to my health than regular cigarettes | **0.32** | **(0.11, 0.95)** | **0.27** | **(0.21, 0.35)** | 0.84 | (0.39, 1.84) |
| E-cigarettes may help me quit smoking cigarettes | 0.45 | (0.15, 1.32) | **0.32** | **(0.25, 0.41)** | 0.97 | (0.46, 2.05) |
| E-cigarette is less toxic than tobacco | **0.31** | **(0.10, 0.94)** | **0.32** | **(0.25, 0.41)** | 0.93 | (0.43, 2.02) |
| Using an e-cigarette feels like smoking a regular cigarette | 0.89 | (0.39, 2.04) | **0.48** | **(0.37, 0.61)** | 1.43 | (0.77, 2.63) |
| People in the media or other public figures use e-cigarettes | 2.71 | (0.65, 11.3) | 1.09 | (0.73, 1.63) | 0.78 | (0.22, 2.83) |
| To avoid bothering others with cigarette smoke | 0.45 | (0.15, 1.32) | **0.34** | **(0.27, 0.44)** | 1.05 | (0.49, 2.21) |
| To completely quit smoking regular cigarettes | 0.44 | (0.15, 1.30) | **0.35** | **(0.27, 0.44)** | 1.34 | (0.63, 2.84) |
| To cut down on the amount of cigarette smoking | 0.45 | (0.15, 1.33) | **0.27** | **(0.21, 0.35)** | 0.98 | (0.45, 2.17) |
| People who are important to me use an e-cigarette | 0.84 | (0.32, 2.21) | 0.77 | (0.54, 1.09) | 0.77 | (0.34, 1.74) |
| E-cigarettes don’t smell | 0.65 | (0.22, 1.92) | **0.32** | **(0.25, 0.41)** | 1.05 | (0.50, 2.18) |
| To avoid having to go outside to smoke | 0.44 | (0.15, 1.31) | **0.39** | **(0.31, 0.50)** | 1.27 | (0.59, 2.75) |
| I like socializing while using an e-cigarette | 1.59 | (0.75, 3.36) | **0.70** | **(0.52, 0.93)** | 1.53 | (0.83, 2.80) |
| I prefer the taste of an e-cigarette | 0.50 | (0.17, 1.49) | **0.41** | **(0.32, 0.53)** | 1.92 | (0.92, 4.01) |
